# Use of Autoantigen-Loaded Phosphatidylserine-Liposomes to Arrest Autoimmunity in Type 1 Diabetes

**DOI:** 10.1371/journal.pone.0127057

**Published:** 2015-06-03

**Authors:** Irma Pujol-Autonell, Arnau Serracant-Prat, Mary Cano-Sarabia, Rosa M. Ampudia, Silvia Rodriguez-Fernandez, Alex Sanchez, Cristina Izquierdo, Thomas Stratmann, Manuel Puig-Domingo, Daniel Maspoch, Joan Verdaguer, Marta Vives-Pi

**Affiliations:** 1 Immunology Department, Germans Trias i Pujol Research Institute, Autonomous University of Barcelona, Badalona, Spain; 2 Catalan Institute of Nanoscience and Nanotechnology, Bellaterra, Spain; 3 Statistics Department, Faculty of Biology, University of Barcelona, Barcelona, Spain; 4 Department of Physiology and Immunology, Faculty of Biology, University of Barcelona, Barcelona, Spain; 5 Endocrinology and Nutrition Service, Hospital Germans Trias i Pujol, Badalona, Spain; 6 Institucio Catalana de Recerca i Estudis Avançats (ICREA), Barcelona, Spain; 7 Immunology Unit, Department of Experimental Medicine, University of Lleida and IRBLleida, Lleida, Spain; University of British Columbia, CANADA

## Abstract

**Introduction:**

The development of new therapies to induce self-tolerance has been an important medical health challenge in type 1 diabetes. An ideal immunotherapy should inhibit the autoimmune attack, avoid systemic side effects and allow β-cell regeneration. Based on the immunomodulatory effects of apoptosis, we hypothesized that apoptotic mimicry can help to restore tolerance lost in autoimmune diabetes.

**Objective:**

To generate a synthetic antigen-specific immunotherapy based on apoptosis features to specifically reestablish tolerance to β-cells in type 1 diabetes.

**Methods:**

A central event on the surface of apoptotic cells is the exposure of phosphatidylserine, which provides the main signal for efferocytosis. Therefore, phosphatidylserine-liposomes loaded with insulin peptides were generated to simulate apoptotic cells recognition by antigen presenting cells. The effect of antigen-specific phosphatidylserine-liposomes in the reestablishment of peripheral tolerance was assessed in NOD mice, the spontaneous model of autoimmune diabetes. MHC class II-peptide tetramers were used to analyze the T cell specific response after treatment with phosphatidylserine-liposomes loaded with peptides.

**Results:**

We have shown that phosphatidylserine-liposomes loaded with insulin peptides induce tolerogenic dendritic cells and impair autoreactive T cell proliferation. When administered to NOD mice, liposome signal was detected in the pancreas and draining lymph nodes. This immunotherapy arrests the autoimmune aggression, reduces the severity of insulitis and prevents type 1 diabetes by apoptotic mimicry. MHC class II tetramer analysis showed that peptide-loaded phosphatidylserine-liposomes expand antigen-specific CD4^+^ T cells *in vivo*. The administration of phosphatidylserine-free liposomes emphasizes the importance of phosphatidylserine in the modulation of antigen-specific CD4^+^ T cell expansion.

**Conclusions:**

We conclude that this innovative immunotherapy based on the use of liposomes constitutes a promising strategy for autoimmune diseases.

## Introduction

An important goal in type 1 diabetes (T1D) prevention is the arrest of the autoimmune reaction to β-cells. The uptake of apoptotic cells by antigen presenting cells, called efferocytosis, is an essential process in immunological tolerance induction [1, 2]. Apoptotic cells exhibit surface changes, especially exposure of the plasma membrane inner leaflet phospholipid phosphatidylserine (PS) that distinguish them from viable cells and allow recognition by efferocytic receptors [3]. Many of these receptors of eat-me signals-TIM-4, CD14, BAI1 and α_v_β_3_ integrin, among others- may contribute to form the engulfment synapse [4]. Defective efferocytosis is associated to T1D and other autoimmune diseases [5, 6]. The efficacy of an antigen-specific cell immunotherapy based on apoptosis in preventing experimental T1D was previously demonstrated [7] and consists in dendritic cells (DCs) loaded with apoptotic β-cells, which reestablishes immunological tolerance through suppressive DCs and prostaglandin E_2_ (PGE_2_) production [8].

Due to the difficulty in obtaining and standardizing β-cell apoptotic bodies, our interest has been drawn to whether peripheral tolerance may be restored by liposomal microparticles containing β-cell antigens, as an alternative source of apoptotic β-cells. Liposomes have the advantages of being easy to prepare, customize and administer, and of constituting a low-cost strategy compared to other current immunotherapies. Moreover, liposomes protect encapsulated antigens from degradation by proteases in the plasma. Lipid-based nanotechnology is an innovative area of great scientific interest that includes liposomes, which are currently used clinically as vehicles for anticancer drugs, vaccines, and in the treatment of autoimmune diseases [9–11].

Several groups have demonstrated that the use of PS-liposomes may inhibit immune responses through down modulation of macrophage and DCs [12–16]. Since antigen specific PS-liposomes resemble apoptotic cells in inhibiting maturation and immunostimulatory function of DCs, these liposomal microparticles could be optimum vehicles to restore tolerance to autoantigens thus preventing the progression of autoimmunity.

## Materials and Methods

### Ethics Statement

*In vivo* experiments were performed in strict accordance with the recommendations in the Guide for the Care and Use of Laboratory Animals of the Generalitat de Catalunya, Catalan Government and the Principles of laboratory animal care (NIH pub.85–23 revised 1985; http://grants1.nih.gov/grants/olaw/references/phspol.htm). The protocol was approved by the Committee on the Ethics of Animal Experiments of the Germans Trias i Pujol Research Institute.

### Mice

Wild-type NOD mice were bred in our own facility and kept under specific pathogen-free conditions, in a 12 h dark/12 h light cycles with food and water *ad libitum*. Only prediabetic 8–12 week old females were included in the study. NOD.Foxp3^EGFP^ mice were generated in-house using a speed congenic approach by backcrossing B6.Foxp3^EGFP^ animals into the NOD background while monitoring 15 independent Idd loci as described [17], and used to study the expansion of Foxp3^+^ CD4^+^ T cells. Mice were sacrificed by cervical dislocation.

### Peptides and liposomes

Insulin peptides were selected because they are target epitopes in autoimmune diabetes [18] and β-cell specific. Peptide A, from insulin A chain (21 aa, N-start-GIVDQCCTSICSLYQLENYCN-C-end) and peptide B, from insulin B chain (30 aa, N-start-FVKQHLCGSHLVEALYLVCGERGFFYTPMS-C-end) were chosen (Genosphere Biotechnologies, Paris, France). The 2.5mi peptide (AHHPIWARMDA), a mimotope for the diabetogenic CD4^+^ T cell clone BDC-2.5, has been described [19] and was selected for tetramer experiments. The peptide derived from glucose-6-phosphate isomerase (GPI) 282–292 (GPI_282–292,_ LSIALHVGFDH) (GL Biochem, Shanghai, China) was used as irrelevant control for tetramer staining [19]. Peptides were >95% pure and Trifluoroacetic acid was removed. Liposomes were composed of 1,2-dioleoyl-sn-glycero-3-phospho-L-serine (sodium salt) (DOPS) and 1,2-didodecanoyl-sn-glycero-3-phosphocholine (DLPC, Lipoid, Steinhausen, Switzerland), and cholesterol (CH, Sigma Aldrich, Saint Louis, MO). Lipid-conjugated fluorescent dye Oregon Green 488 1,2-dihexadecanoyl-sn-glycero-3-phosphoethanolamine (DHPE) was purchased from Invitrogen (Carlsbad, CA). Alexa Fluor 750 was obtained from Invitrogen and was conjugated with the lipid 1,2-Dioleoyl-*sn*-glycero-3-phosphoethanolamine (Avanti Polar Lipids, Alabaster, AL). The liposomes were prepared using the thin film hydration method from a lipid mixture of PS, PC and CH at 1:1:1.33 molar ratio, respectively [15], under sterile conditions. The final concentration of lipid was 30 mM. Lipids and lipid-conjugated fluorescent dyes were dissolved in chloroform and the solvent was removed by evaporation under vacuum and nitrogen. The lipids were hydrated with the appropriate buffer (PBS, 0.5mg/mL solution of insulin peptide A, insulin peptide B or 2.5mi peptide) and the liposomes obtained were homogenized to 1 μm by means of an extruder (Lipex Biomembranes, Vancouver, Canada). Encapsulation efficiencies (EE) were calculated according to the equation EE(%) = [(C_peptide,total_-C_peptide,out_)/C_peptide,total_] x100, where C_peptide,total_ is the initial peptide A or B concentration and C_peptide,out_ is the concentration of non-encapsulated peptide. To measure the C_peptide,out_, all liposome suspensions were centrifuged at 110000g for 30 min at 10°C. The concentration of non-encapsulated peptide was assessed in supernatants by PIERCE BCA protein assay kit (Thermo Fisher Scientific Inc., Rockford, IL). In addition to PS-liposomes loaded with insulin peptides (PSAB-lipo) or 2.5mi peptide (PS2.5mi-lipo), empty liposomes were generated as controls (PS-lipo). Moreover, liposomes with PC and CH but without PS were generated as controls, empty (PC-lipo) or loaded with 2.5mi peptide (PC2.5mi-lipo), following the same methodology used in PS-liposomes. Particle size distributions and stability of liposomes-expressed as zeta potential (ζ)- were measured by dynamic light scattering (DLS) using Malvern Zetasizer (Malvern Instruments, UK) in undiluted samples. The morphology and lamellarity of liposomes were examined using cryogenic transmission electron microscopy (cryo-TEM) in a JEOL-JEM 1400 microscope (Jeol Ltd., Tokyo, Japan).

### Dendritic cells and uptake of liposomes

DCs were generated from bone marrow progenitor cells in culture medium with GM-CSF (1000 U/ml; Prospec, Rehovot, Israel) [7]. Control DCs were either cultured in basal conditions to obtain immature DCs (iDCs) or with lipopolysaccharide (LPS, 100ng/ml; Sigma) for 24 hours to obtain mature DCs (mDCs). DCs purity was assessed by CD11c-PECy7 staining (BD Biosciences, San Jose, CA). Cell viability was determined by annexin V-phycoerythrin (PE) and 7aad staining (BD Biosciences) and cells were counted by flow cytometry (Perfect Count Microspheres, Cytognos, Salamanca, Spain). Preliminary experiments were performed to define optimal conditions. To determine whether liposome uptake by DCs occurs through phagocytosis, *in vitro* assays were performed by coculturing DCs with 100 μM fluorescence labeled liposomes (Oregon green 488 DHPE, Invitrogen) during 5 min to 4 hours at 37°C and at 4°C. After extensively washing in PBS to remove the liposomes attached to the cell membrane, liposome capture was determined by flow cytometry (FACSCanto II, BD Biosciences).

### Flow cytometry

The expression of costimulatory molecules CD40 and CD86 was assessed in the membrane of DCs by flow cytometry (FACSCanto II). DCs were cocultured with 1mM liposomes during 2 hours and maintained in basal conditions or matured with 100 ng/ml LPS (Sigma) for additional 24 hours. DCs were then stained with monoclonal antibodies to mouse CD11c-PECy7, CD40-allophycocyanin (APC), CD86-PE (BD Biosciences), class I MHC H-2K^d^-eFluor450 and class II MHC I-A^d^-APC (eBioscience, San Diego, CA). Corresponding fluorescence minus one (FMO) staining was used as control. Data were analyzed using FlowJo software (Tree Star, Ashland, OR).

### ELISA

The production of PGE_2_ was assessed by ELISA (PGE_2_ EIA Kit-Monoclonal; Cayman Chemicals, Ann Arbor, MI), in supernatants obtained from cell cultures, 24 hours after liposome capture by DCs. Limit of detection: 80% B/B_0_: 15 pg/ml. Sensitivity: 50% B/B_0_: 50 pg/ml. Results were expressed as pg of PGE_2_/10^6^ cells.

### T cell proliferation assays and cytokine production

DCs were loaded with 1 mM liposomes (empty or loaded with insulin peptides) during 2 hours in the presence of insulin (20μg/ml, Sigma). DCs were cultured in basal conditions or matured with LPS (100 ng/ml, Sigma) for additional 24 hours to determine tolerogenic function stability. T cells were obtained after mechanical disruption of spleen and purified by negative selection using antibodies to CD19-PE, CD16/32-PE, CD11c-PECy7 (BD Biosciences), CD11b-PE (ImmunoTools GmbH, Friesoythe, Germany), and sorted (FACSAria II, BD Biosciences) as previously described [8]. 10^4^ DCs were then cocultured with 10^5^ T lymphocytes (1:10 ratio). As a control, T lymphocytes (10^5^) were cultured in basal conditions. After 5 days, cells were pulsed with 1 μCi of (^3^H)-thymidine (Perkin Elmer, Waltham, MA) for an additional 16 hours. Cells were harvested (Harvester 96, Tomtec Inc., Hamden, CT) and analyzed using a scintillation counter (1450 Microbeta, TriluxWallac, Turku, Finland). T cell proliferation was expressed as counts per minute (c.p.m). Cytokine production was assessed using The Mouse Th1/Th2/Th17 kit (CBA system; BD Biosciences) in supernatants from proliferation assays. Data were analyzed using CBA software. The production of TGF-β was determined using Human/Mouse TGF-β1 Ready-SET-Go! (eBioscience).

### *In vivo* tracking of liposomes by bioimaging

*In vivo* and *ex vivo* near-infrared fluorescence imaging was performed using the Pearl Impulse imaging system (LI-COR, Lincoln, NE). NOD mice were anesthetized with ketamine/xylazine at 50 and 5 mg/kg body weight, respectively. *In vivo* imaging was performed at 6, 24, and 48 hours after intraperitoneal (i.p.) administration of 3.5 mg of fluorescence labeled PS-liposomes (Alexa Fluor 750, Invitrogen) in 200 μl of saline solution. At the end of every checkpoint, perigonadal adipose tissue, kidney, spleen, pancreas, pancreatic lymph nodes, mesenteric lymph nodes, liver, mediastinal lymph nodes and thymus were harvested, washed in PBS, and imaged *ex vivo* using the Pearl Impulse system (LI-COR). Fluorescent signal intensity was semi-quantitatively assessed: Fluorescence level was normalized by subtracting the background and represented as a relative index of fluorescence in each organ (RFU) / grams of tissue.

### Type 1 diabetes prevention and insulitis score

NOD mice at 8 weeks of age-normoglycemic and without clinical symptoms of the disease- were treated with a single i.p. dose of 3.5 mg of PS-liposomes (empty or peptide-filled) in 200 μl saline solution. A sham-control group, which only received saline solution, was also included. A minimum of 12 animals per group was analyzed. Mice were monitored daily for urine glucose using Glucocard strips (Menarini, Barcelona, Spain), and weekly for body weight until 30 weeks of age. Mice with glycosuria were confirmed diabetic when the blood glucose level was >300 mg/dl. Insulitis score was determined at the end of the study in all non-diabetic mice. Briefly, pancreata were snap frozen in an isopentane/cold acetone bath. Cryosections of 5 μm were obtained at non-overlapping levels, stained with hematoxylin and eosin, and analyzed by two independent observers. A minimum of 40 islets per animal was analyzed. Insulitis was scored as described elsewhere [20]: 0, no insulitis; 1, peri-insular; 2, mild insulitis (<25% of the infiltrated islet); 3, 25–75% of the islet infiltrated; 4, >75% islet infiltration.

### *In vivo* antigen specific assay and MHC class II tetramer analysis

Prediabetic NOD and NOD.Foxp3^EGFP^ mice were treated with a single 3.5 mg i.p. dose of 2.5mi peptide-loaded PS-liposomes (PS2.5mi-lipo) in 200 μl saline solution. Control groups were treated with empty PS-liposomes, 2.5mi peptide-amount calculated based on encapsulation efficiency- or saline solution (sham). Moreover, PS-free liposomes, empty (PC-lipo) and loaded with 2.5mi peptide (PC2.5mi-lipo), were also administered as controls. At day 4 after the treatment, spleen, pancreatic lymph nodes and mediastinal lymph nodes were harvested and cell suspension was obtained through mechanical disruption. Soluble A^g7^ MHC molecules complexed to the 2.5mi or the GPI control peptide were expressed in *Drosophila melanogaster* derived SC2 cells, transfected with DNA plasmids coding for the A^d^ α-chain containing a biotinylation sequence and the A^g7^ β-chain connected N-terminally to 2.5mi or GPI_282–292_. Molecules were purified and biotinylated using the BirA enzyme (Avidity, CO). Tetramers were generated by incubation of A^g7^ monomers with PE-labeled streptavidin (Columbia Bioscience, Columbia, MD) in a 5:1 ratio [19, 21, 22]. Antigen-specific T cell analysis was performed as previously described [19]. Briefly, single-cell suspension was blocked with avidin (Sigma) in PBS containing 2% FBS and 0.04% NaN_3_, and stained with PE-labeled MHC-peptide tetramers. Antibodies to CD4-pacific blue (PB), CD8-PECy5, CD19-PECy5, F4/80-PECy5 and CD11c-PECy5 were used (BioLegend, CA). Dead cells were excluded with addition of propidium iodide (PI). Flow cytometry was carried out using a FACSCanto II (BD Biosciences) and data were analyzed using FlowJo software (Tree Star).

### Statistical analysis

Statistical analysis was performed using the Prism 5.0 software (GraphPad software Inc., San Diego, CA), SPSS Statistics 17.0 software (SPSS Inc. Chicago, IL) and “R” software (www.r-project.org). The analysis of variance (ANOVA) was used for comparisons with several factors. For paired comparisons, a non-parametric Wilcoxon test was used. Otherwise, Mann Whitney test was applied. For single comparisons, a p-value ≤ 0.05 was considered significant. When several related comparisons were done, a Bonferroni adjustment was performed. Kaplan-Meier log-rank analysis was used for comparisons between survival curves.

## Results

### Phosphatidylserine (PS)-presenting liposomes filled with insulin peptides are captured by DCs

**Fig 1:**
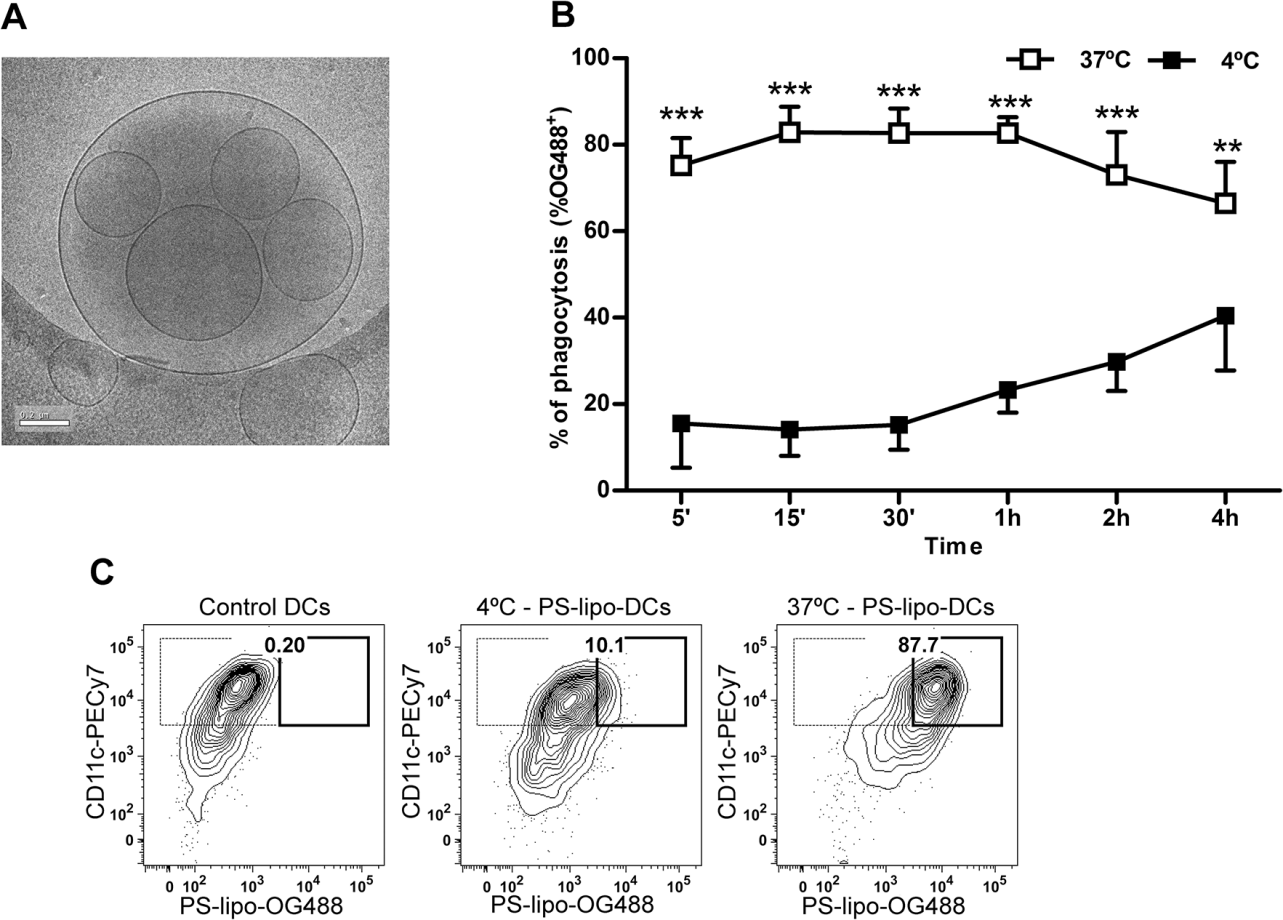
Liposome features. A) Cryogenic transmission electron microscopy images of PSAB-liposomes. Bar = 0.2 μm. B) Time course analysis of the capture of 100 μM OG488 labeled PS-liposomes (OG488 PS-liposome) by DCs at 37°C (white squares) and at 4°C (black squares). Results are expressed as mean±SD of three independent experiments (***p<0.001, **p<0.01, Two-way ANOVA). C) Flow cytometry contour plots of the uptake of PS-liposomes (OG488+) by DCs (CD11c+). From left to right, control DCs, DCs cocultured with OG488 PS-liposome at 4°C and at 37°C. One representative experiment of three is shown. Percentage of liposome capture (thick line) is referred to CD11c+ cell subset (thin line).

PS-liposomes were prepared with DOPS/DLPC/CH at 1:1:1.33 molar ratio to present PS on their surface. Empty liposomes presented a mean diameter of 996.71±89.42 nm (mean±SD) with a polydispersity index (PdI) of 0.31±0.05. Zeta potential measurements revealed a net surface charge of -29.26±2.82 mV on PS-liposomes. When PS-liposomes were loaded with peptide A or B from mouse Insulin2, the mean diameter was 1051.43±45.15 nm (PdI = 0.31±0.06) and 968.57±86.32 nm (PdI = 0.27±0.08), respectively. Encapsulated liposomes had a negative surface charge of -30.79±2.35 mV for peptide A and -29.44±1.48 mV for peptide B. The mean of percentage of encapsulation was 41.07±23.58% for peptide A and 87.44±4.54% for peptide B (Table 1). Cryo-TEM analysis revealed a multivesicular vesicle morphology (Fig 1A).

**Table 1:** Insulin-loaded liposome characteristics.

Liposome type	Diameter (nm)	Polydispersity index (PdI)	Zeta potential (mV)	Encapsulation efficiency (%)
**PS-liposomes**	996.71 ± 89.42	0.31 ± 0.05	-29.26 ± 2.82	-
**PSA-liposomes**	1051.43 ± 45.15	0.31 ± 0.06	-30.79 ± 2.35	41.07 ± 23.58
**PSB-liposomes**	968.57 ± 86.32	0.27 ± 0.08	-29.44 ± 1.48	87.44 ± 4.54

Data are expressed as mean±SD.

Time course analysis (Fig 1B) confirmed that 75.20±6.38% of phagocytosis was achieved after 5 min of coculture at 37°C. As expected, OG488 signal was significantly higher (p<0.001, and p<0.01) in each checkpoint when compared to the same experiment performed at 4°C. Fig 1C shows liposome capture by DCs after 30 min of coculture at 37°C. We observed greatly decreased signal in experiments performed at 4°C, confirming the capture of liposomes by phagocytosis.

### The capture of PS-liposomes by iDCs induces a semimature phenotype

**Fig 2:**
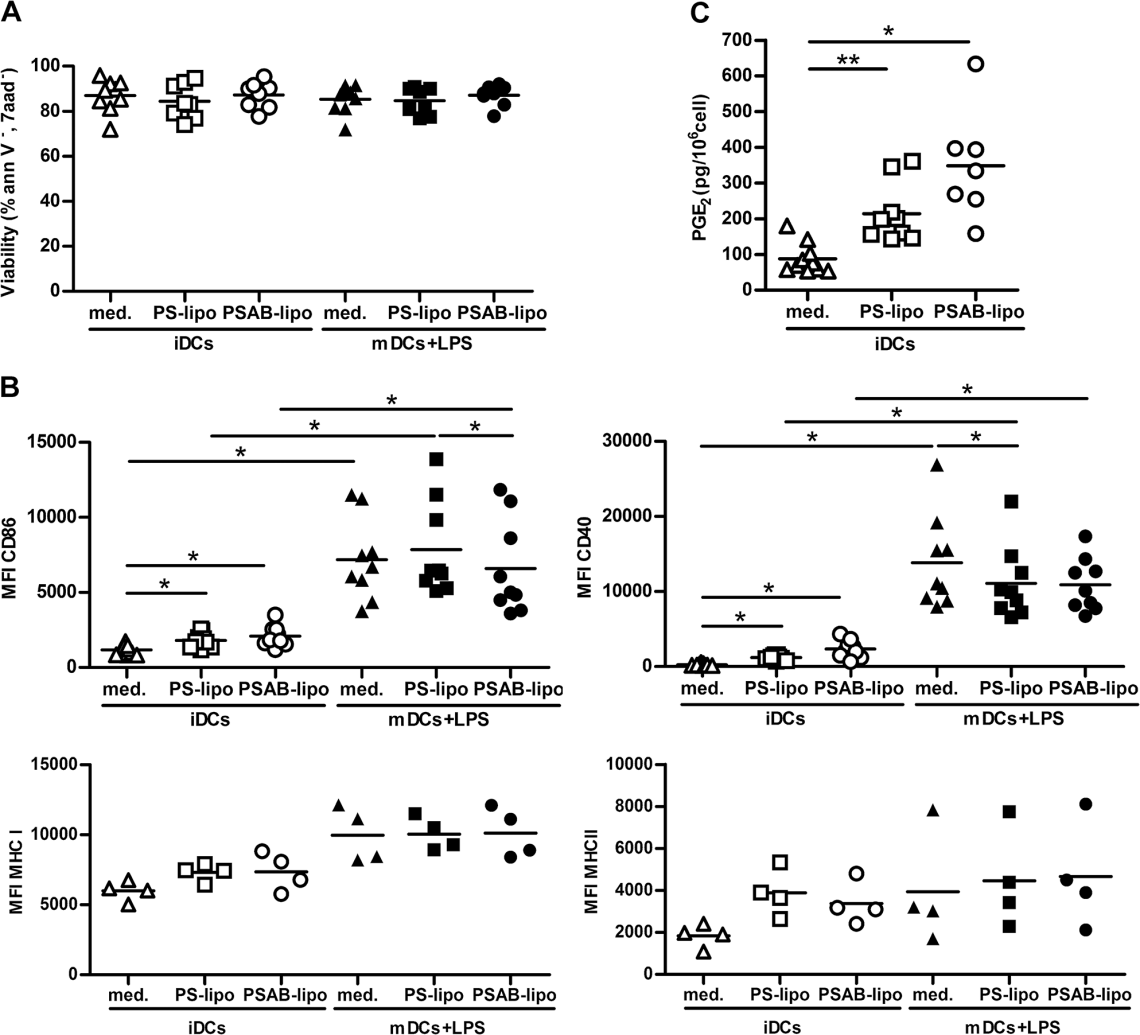
Effects of the capture of PS-liposomes in DCs phenotype. A) DCs viability assessed by annexin V and 7aad staining. White symbols represent iDCs, before (triangles) and after the capture of PS-liposomes (squares) or PSAB-liposomes (circles), 24 hours after culture. Black symbols represent viability of mature DCs (mDCs) before (triangles) and after the capture of PS-liposomes (squares) or PSAB-liposomes (circles) after proinflammatory stimulus (LPS). Lines show the mean of at least eight independent experiments. B) Median of fluorescence intensity (MFI) for CD86, CD40, MHC Class I and MHC Class II membrane expression on DCs before and after liposome capture (white symbols) and after exposure to LPS (black symbols). Lines show the mean of at least four independent experiments. Comparisons within each group and between paired maturation conditions showed significant differences (*p<0.05, Wilcoxon test). C) Quantification of the PGE_2_ production by immature DCs (iDCs) in culture medium (med, white triangles), loaded with PS-liposomes (PS-lipo, white squares) or PSAB-liposomes (PSAB-lipo, white circles), after 24 hours of culture. Data are represented as pg/10^6^ cells. Lines show the mean of a minimum of seven independent experiments. Comparisons between groups showed significant differences (**p<0.01 and *p<0.05, Wilcoxon test).

Viability of DCs after coculture with liposomes was always similar to control DCs even after a proinflammatory stimulus (Fig 2A), regardless of the dose (range 100–1000 μM, data not shown). We next examined whether liposome capture affected the expression of membrane MHC and costimulatory molecules (Fig 2B). The expression of CD86 and CD40 in iDCs membrane slightly increased after PS- or PSAB-liposome capture (p<0.05), suggesting that iDCs had acquired a semimature phenotype, a feature of tolerogenic DCs. After LPS exposure, control DCs significantly increased the membrane expression levels of costimulatory molecules and activation markers CD40 and CD86 (p<0.05). Also, after the addition of LPS, DCs loaded with PS- or PSAB-liposomes significantly increased CD86 and CD40 when compared to their immature counterparts (p<0.05) (Fig 2B). Comparisons within mDCs group showed that CD86 expression was lower in mDCs loaded with PSAB-liposomes than PS-liposomes pulsed mDCs (p<0.05). Also, CD40 expression was lower in mDCs loaded with PS-liposomes when compared to unloaded mDCs (p<0.05). PS- or PSAB-liposome capture did not significantly increase the expression of MHC in iDCs. However, a tendency to increase MHC class I and class II expression was observed after the capture of PS- or PSAB-liposomes by iDCs, and after LPS exposure (Fig 2B).

Based on our previous results [8], PGE_2_ production by DCs after PS-liposome uptake was measured. The concentration of PGE_2_ was significantly increased in the supernatant of iDCs cocultured with PS-liposomes (p<0.01) or PSAB-liposomes (p<0.05) when compared to iDCs (Fig 2C).

### Impairment of DCs to stimulate autologous T cell proliferation after PS-liposome uptake

**Fig 3:**
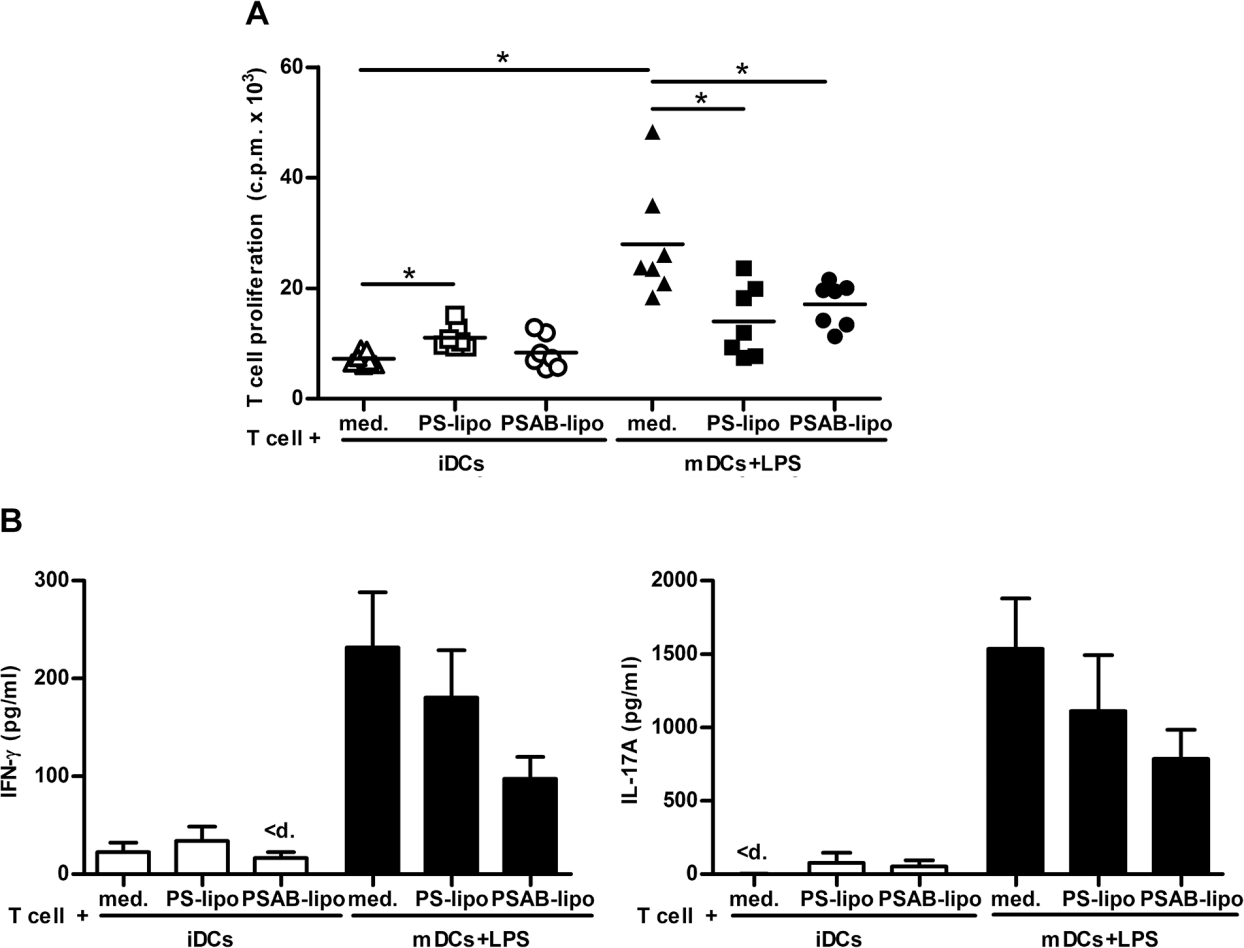
Impaired DCs’ ability to induce autologous T cell proliferation after PS-liposomes capture, and cytokine secretion. A) Autologous proliferation of T cells (c.p.m. for ^3^H thymidine assay) after stimulation induced by iDCs, before (white triangles) and after the capture of PS-liposomes (white squares) or PSAB-liposomes (white circles), with insulin (20 μg/ml) at a ratio of 1:10 for 6 days. Black symbols represent proliferation induced by mature DCs (mDCs) before (triangles) and after the capture of PS-liposomes (squares) or PSAB-liposomes (circles), previously activated with proinflammatory stimuli LPS (100 ng/ml). Lines show the mean of seven independent experiments. Comparisons within each group and between paired maturation conditions showed significant differences (*p<0.05, Wilcoxon test). B) Cytokine production in T cell proliferation experiments. Levels of IFN-γ and IL-17A were measured in supernatants from T cell proliferation experiments induced by iDCs, DCs loaded with empty liposomes (PS-lipo) and DCs loaded with liposomes with insulin peptides (PSAB-lipo) in basal conditions (white bars) or after 24 hours with LPS (black bars). Results are expressed as mean±SEM from four independent experiments. Comparisons within each group and between paired maturation conditions were not able to detect significant differences (p<0.05, Wilcoxon test). “<d” means values below the standard.

DCs derived from bone marrow progenitor cells were >80% pure (CD11c+). Viability was always >90%. T cell purity and viability were always over 85% and 90% respectively (S1 Fig). The capture of PSAB-liposomes by iDCs did not increase autologous T cell proliferation when compared to iDCs (Fig 3A). A slight increase in T cell proliferation was observed after the capture of empty PS-liposomes (p<0.05). As expected, T cell proliferation induced by mDCs was higher than proliferation induced by iDCs (p<0.05). Interestingly, T cell proliferation induced by mDCs loaded with PS- or PSAB-liposomes was significantly lower than proliferation induced by unloaded mDCs (p<0.05), even after LPS stimulus, showing the functional effect of liposomes on mDCs. Predictably, purified T cells cultured without DCs did not show proliferation (0.07±0.03 c.p.m. x 10^3^, mean±SD).

### The uptake of autoantigen-loaded liposomes by iDCs does not alter cytokine profile

T cells cocultured with iDCs loaded with PS- or PSAB-liposomes displayed a cytokine profile (IFN-γ, IL-17A) similar to unloaded iDCs (Fig 3B). After LPS stimulus, T cells cocultured with mDCs or mDCs loaded with PS- or PSAB-liposomes display a tendency to increase the secretion of IFN-γ and IL-17A when compared to their iDCs counterparts. However, a trend toward lower IFN-γ and IL-17A secretion was observed in PSAB-loaded mDCs condition compared with unloaded mDCs. No differences were observed in IL-6, TNF and TGF-β secretion by iDCs and mDCs due to the uptake of PS- or PSAB-liposomes. IL-2, IL-10 and IL-4 were not detected in any condition of the assay (data below the detection limit) (S2 Fig).

### *In vivo* tracking of liposomes

**Fig 4:**
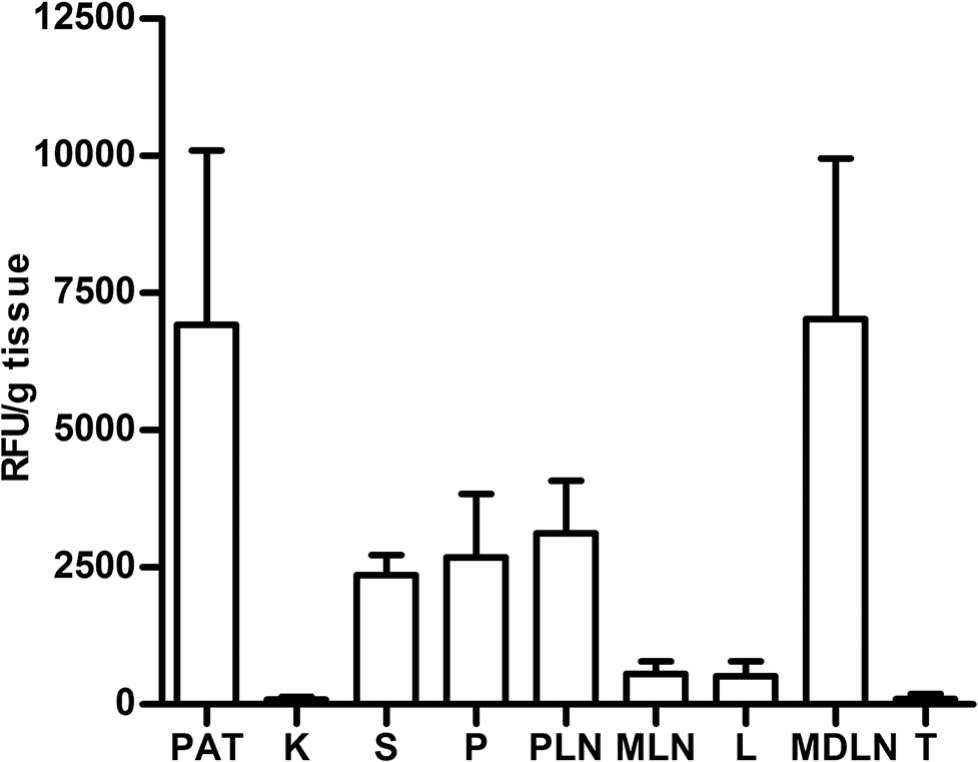
Tracking of PS-liposomes. Histogram of *ex vivo* relative fluorescent signal (RFU, Relative Fluorescence Units/g tissue) in organs from NOD mice 24 hours after the administration of labeled PS-liposomes (Alexa Fluor 750). PAT, perigonadal adipose tissue; K, kidney; S, spleen; P, pancreas; PLN, pancreatic lymph nodes; MLN, mesenteric lymph nodes; L, liver; MDLN, mediastinal lymph nodes; T, thymus. Results are mean±SEM of three independent experiments.

To determine liposomes biodistribution, fluorescence labeled PS-liposomes were i.p. administered to NOD mice. The *ex vivo* analysis of the organs showed that, 24 hours post administration, fluorescent signal took place primarily in perigonadal adipose tissue, mediastinal lymph nodes, pancreatic lymph nodes, pancreas, spleen, mesenteric lymph nodes and liver, whereas the minimum signal was detected in kidney and thymus (Fig 4). Only traces of fluorescent signal were detected in lungs.

### Insulin peptide-filled PSAB-liposomes decrease T1D incidence and reduce insulitis in NOD mice

**Fig 5:**
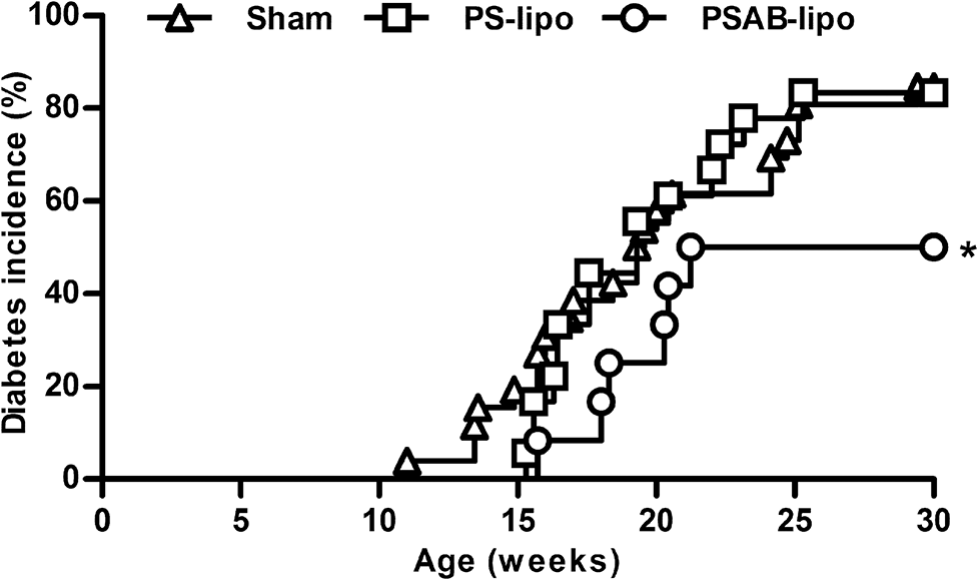
Immunotherapy using PS-liposomes filled with insulin peptides decreases T1D incidence. Cumulative incidence (percentage) of T1D in NOD mice treated with PSAB-liposomes (PSAB-lipo, circles, n = 12), PS-liposomes (PS-lipo, squares, n = 18), and sham group (triangles, n = 26). Significant differences were found when compared group treated with PSAB-liposomes *versus* sham group (*p≤0.05, Kaplan-Meier log-rank analysis).

**Fig 6:**
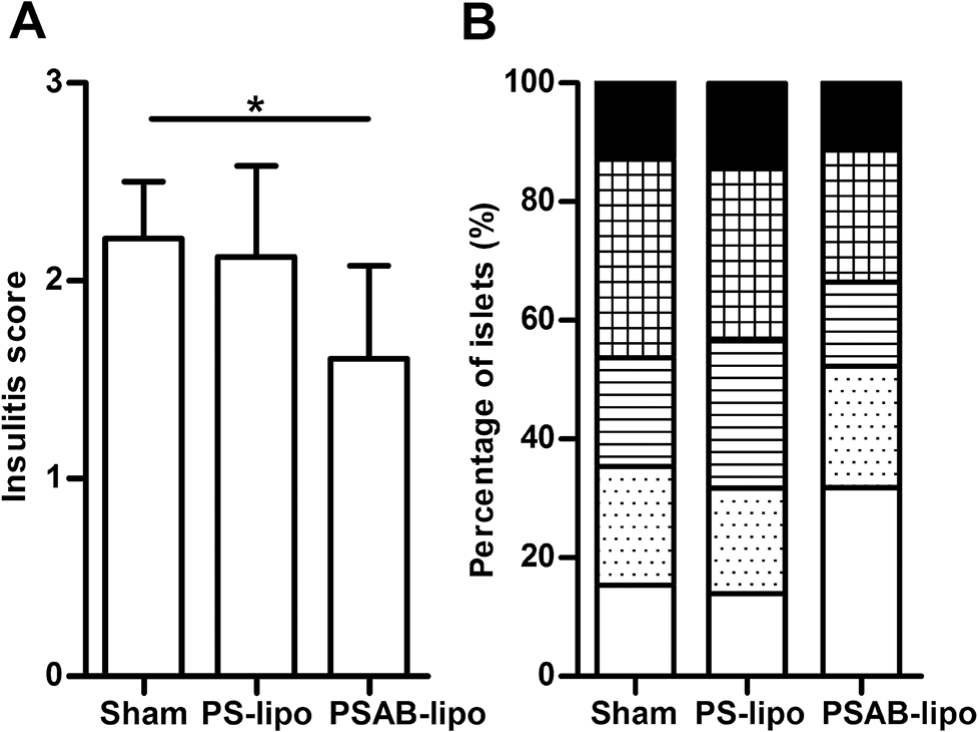
Reduction in insulitis following administration of PSAB-liposomes. A) Insulitis score from non-diabetic mice at the end of the follow-up (30 weeks), sham (n = 4), PS-liposomes (PS-lipo, n = 3) and PSAB-liposomes containing autoantigens (PSAB-lipo, n = 6). Results are mean±SD (*p≤0.05, Mann Whitney test). B) Percentage of islets in each of the infiltration categories: White = 0, no insulitis; Dotted = 1, peri-insular; Striped = 2, mild insulitis (<25% of the infiltrated islet); Squared = 3, 25–75% of the islet infiltrated; Black = 4, >75% islet infiltration.

NOD mice were treated with a single dose of immunotherapy during the prediabetic period (8 weeks old). No significant differences were found in body weight in mice treated with PSAB-liposomes, PS-liposomes, or saline solution (data not shown). As expected, animals from the sham-control group developed diabetes from the age of 11 weeks and with a final incidence of 84.62% (Fig 5). The treatment with empty PS-liposomes resulted in a disease incidence of 83.33% starting the disease at 15 weeks of age. No significant differences were found between both groups. Mice treated with PSAB-liposomes containing β-cell autoantigens showed a T1D incidence of 50%, significantly lower than sham group (p≤0.05) and slightly delayed, starting at 16 weeks of age. Insulitis score was determined at the end of the follow-up period rid="_Ref959725601" (Fig 6A) As expected, mice in the sham group showed a high degree of insulitis score (2.22±0.29, mean±SD). Mice treated with PS-liposomes showed a similar insulitis degree (2.12±0.46) than sham group. Mice treated with PSAB-liposomes displayed a significant reduction in insulitis (1.61±0.47) when compared to the sham group (p<0.05) and, a biological reduction, although non-significant, when compared to mice treated with PS-liposomes. In mice treated with PSAB-liposomes, 52.31% of the islets remained non-destructed-insulitis free or with peri-insulitis-, whereas 35.39% and 31.69% of the islets were non-destructed in the sham and the PS-liposomes groups, respectively (Fig 6B)

### Autoantigen-loaded liposomes induce an antigen-specific T cell response

To test how peptide-loaded PS-liposomes affected antigen-specific CD4^+^ T cells *in vivo*, we carried out tetramer staining experiments. As functional A^g7^/insulin peptide tetramers were not available, we used the 2.5mi peptide instead. 2.5mi is a strong mimotope agonist peptide for the diabetogenic CD4^+^ T cell clone BDC-2.5 [23, 24], and A^g7^/2.5mi tetramers enable the detection of a natural CD4^+^ T cell population, termed 2.5mi^+^ T cells, that share antigen recognition with this T cell clone in NOD mice [19]. PS2.5mi-liposomes presented a mean diameter of 875.37±63.27 nm (mean±SD) with a PdI of 0.35±0.13. Zeta potential measurement revealed a net surface charge of -32.60±2.18 mV. The mean of percentage of encapsulation was 22.94±12.72%. Empty PS-liposomes were used as controls as described above. Additional controls were empty PC-liposomes and PC2.5mi-liposomes. Empty PC-liposomes showed a mean particle size of 1971.50±895.90 nm with a PdI of 0.69±0.44 and a net surface charge of -4.34±0.07mV. PC2.5mi-liposomes presented a mean particle size of 2055.33±353.23 nm with a PdI of 0.35±0.17, a net surface charge of -4.52±0.34mV and a percentage of encapsulation of 16.68±2.90. This low encapsulation efficiency was due to the low affinity of the positively charged peptide to a much less negatively charged PS-free liposome, in comparison to PS-liposomes, as described [25].

**Fig 7:**
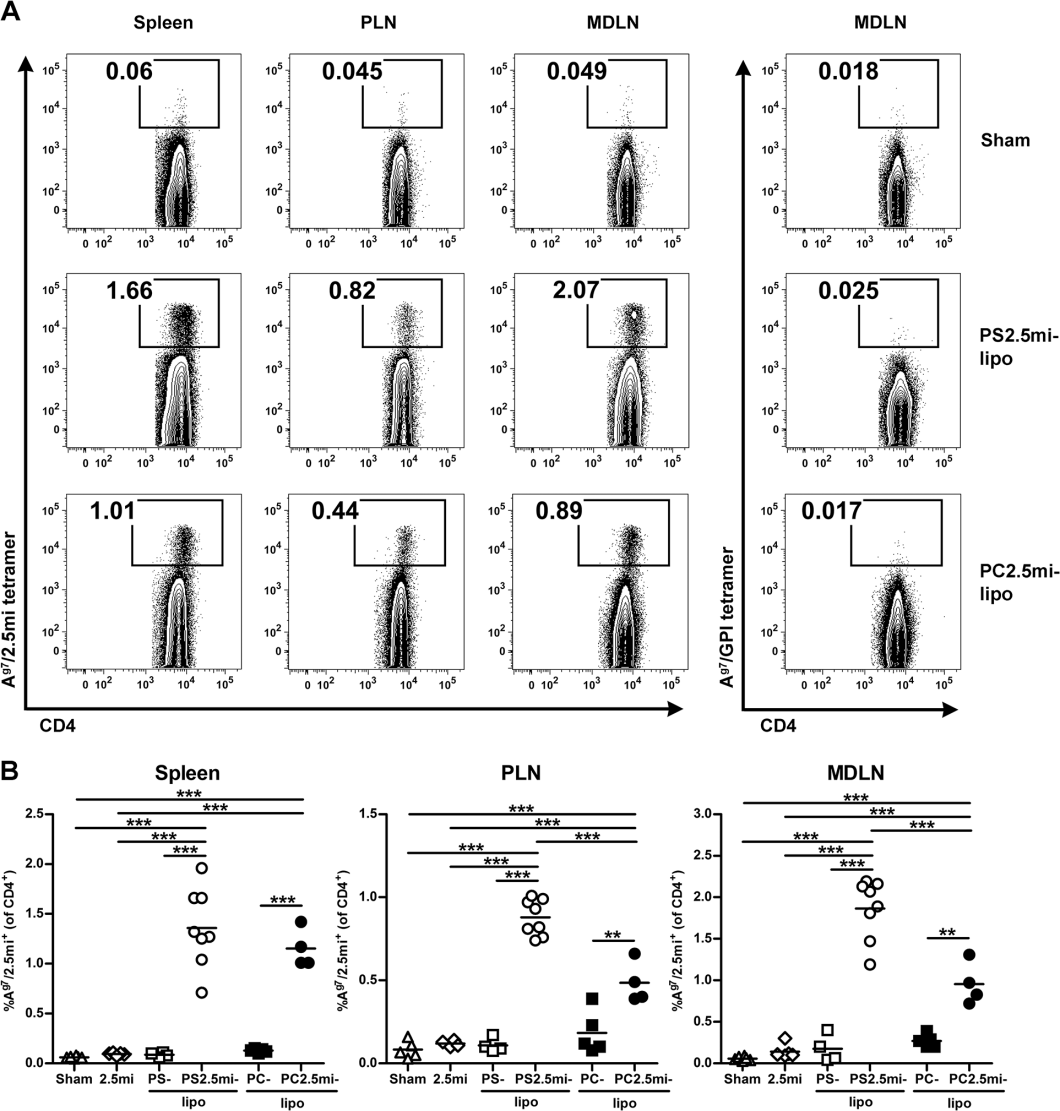
Antigen-specific CD4^+^ T cell expansion 4 days after i.p. administration of PS2.5mi-liposomes to NOD mice. A) Representative flow cytometry contour plots of the percentage of 2.5mi^+^ CD4^+^ T cells in the spleen, pancreatic lymph nodes (PLN) and mediastinal lymph nodes (MDLN) (gated on CD19^-^, CD8^-^, F4/80^-^, CD11c^-^, PI^-^ and CD4^+^ cells) in the sham group and after the administration of PS2.5mi-liposomes or PC2.5mi-liposomes. Left panel: A^g7^/2.5mi tetramer staining. Right panel: control staining with A^g7^/GPI_282–292_ tetramer. B) Detection of 2.5mi^+^ CD4^+^ T cells in the spleen, PLN and MDLN from 4–8 mice pooled from 4 independent experiments after the administration of saline solution (white triangles, n = 6), 2.5mi peptide (white rhombus, n = 5), PS-liposomes (white squares, n = 4), PS2.5mi-liposomes (white circles, n = 8), PC-liposomes (black squares, n = 5) or PC2.5mi-liposomes (black circles, n = 4). Comparisons between groups showed significant differences (***p<0.001, One-way ANOVA and Bonferroni multiple comparison test).

The treatment with one dose of PS2.5mi-liposomes significantly expanded antigen-specific CD4^+^ T cells (p<0.001)-at day 4 after the i.p. injection- when compared to treatment with saline solution, 2.5mi peptide or PS-liposomes (Fig 7). This effect was detected in the spleen, pancreatic lymph nodes and mediastinal lymph nodes. PC2.5mi-liposomes induced a lower response of antigen-specific CD4^+^ T cells than PS2.5mi-liposomes in PLN and MDLN (Fig 7).

We next examined the generation of antigen-specific Foxp3^+^ CD4^+^ T after PS2.5mi-liposome administration using NOD.Foxp3^EGFP^ reporter mice. It has been shown that these mice generate 2.5mi^+^ T cells as well as Foxp3^+^ Treg with the same specificity [17]. Antigen-specific CD4^+^ T cells, both Foxp3^-^ T cells and Foxp3^+^ T cells, were expanded in the spleen, PLN and MDLN from mice treated with PS2.5mi-liposomes (S3 Fig). A 29–36 fold stronger antigen-specific CD4^+^ Foxp3^-^ T cell proliferation, and a 4–17 fold stronger antigen-specific CD4^+^ Foxp3^+^ T cell proliferation were detected after PS2.5mi-liposomes treatment, when compared to sham group (S3B Fig). Taken together, our results show that PS2.5mi-liposome administration favors antigen-specific CD4^+^ T cell expansion, observed in the Foxp3^+^ as well as in the Foxp3^-^ T cell compartments.

## Discussion

Apoptotic cells, when engulfed by antigen presenting cells, contribute to the maintenance of self-tolerance [26]. Based on this feature, we have designed a synthetic strategy to arrest autoimmunity against β-cells, consisting in liposomal microparticles designed to mimic apoptotic cells in terms of PS recognition and filled with insulin peptides. These liposomes prevent T1D through specific reestablishment of tolerance.

Indeed, we have previously demonstrated that the tolerance to β-cells is restored by the administration of tolerogenic DCs loaded with apoptotic β-cells [7], through tolerogenic features and suppressive effects acquired by DCs after efferocytosis [8]. Due to the difficulty in obtaining, preserving and standardizing apoptotic cells, an important issue was: how could apoptotic β-cells be mimicked for therapeutic purposes? A central event of apoptotic cells is the loss of phospholipid arrangement and the exposure of phosphatidylserine component, which is present in the inner layer of the cell membrane and will provide the main signal for efferocytosis. Therefore, PS-liposomes filled with insulin peptides were prepared, in the size range for an efficient phagocytosis [27, 28] and with multivesicular vesicles morphology, with advantages in terms of encapsulation [29] and immunological activity [30]. Several groups have demonstrated the anti-inflammatory effects of PS-liposomes [12–15]. This feature is dependent on the exposure of phosphatidylserine [16, 31] and mimics apoptotic cell clearance, an immunologically silent event [32]. Anionic liposomes, composed of phosphatidylserine, phosphatidylcholine, and cholesterol, interact with DCs promoting phagocytosis [33]. Therefore, PS-liposomes could be optimum vehicles of β-cell autoantigens, by simulating apoptotic cell recognition and inhibiting maturation and immunostimulatory function of DCs [34].

Insulin is an obvious target for antigen specific interventions in humans and NOD mice. Oral administration of insulin resulted in disease prevention in animal models [35, 36], but in humans, the results have not been very encouraging. An added benefit of liposome approach is the delivery of insulin peptides along with PS, a combination that can contribute to a stable and long-term specific tolerance reestablishment. To avoid possible biological effects of insulin, we decided to encapsulate the peptides corresponding to the whole A and B chain of insulin. These two peptides contain well-known β-cell specific target epitopes in T1D [18, 35, 37]. Peptide A showed a reduction in the encapsulation when compared to peptide B, a difference attributed to aminoacid composition and peptide solubility. To improve this immunotherapy, additional T1D autoantigens should be evaluated for encapsulation in terms of solubility, size and antigenicity [38]. After liposome engulfment by DCs, autoantigens would be presented to CD4^+^ T lymphocytes through MHC class II molecules and to CD8^+^ T lymphocytes by cross presentation [39] in tolerogenic forms [40, 41].

*In vitro* experiments revealed important immunological features. First, PS- and PSAB-liposomes were safely phagocytosed by DCs because liposomes do not degenerate into toxic side products, such as necrotic bodies. Second, after liposome uptake, iDCs slightly increased CD86, CD40 membrane expression and showed a biological, although non significant, increase of MHC class II expression. This phenotype suggests that DCs acquired a semimature phenotype after liposome capture, a feature of tolerogenic DCs [42]. Moreover, after the uptake of PS- or PSAB-liposomes, DCs were poor stimulators of autologous T cell proliferation, even after proinflammatory stimulus. Third, the liposome uptake induced PGE_2_ secretion by DCs, a tolerogenic mediator of antigen presenting cells [8, 43, 44]. It is well known that the production of PGE_2_ by PS-liposomes depends on the exposure of PS and ligation of its receptor; in fact, PGE_2_ secretion by DCs is not induced by phosphatidylcholine liposomes [14, 45]. And finally, given the role of IL-17 and IFN-γ in β-cell apoptosis and T1D [46], we showed that IL-17A and IFN-γ production was not increased in T cell proliferation assays induced by DCs after autoantigen-loaded liposome uptake. Also, a trend toward lower cytokine secretion was observed even after a proinflammatory stimulus, suggesting that PSAB-liposomes contribute to tolerance reestablishment, by antigen specific memory cells. Taken together, these results suggest that PSAB-liposomes may inhibit the DCs’ ability to stimulate T cell response and may promote tolerance, similar to that induced by apoptotic cells in an antigen-specific manner [8]. We are well aware that liposome design and administration pattern should be improved to better simulate apoptotic β-cells. In this sense, liposomes could be customized, encapsulating peptides from other T1D autoantigens, thus inactivating the autoreactive T cell repertoire. Also, the administration pattern must be optimized to improve the effect of the immunotherapy.

Another important issue for this immunotherapy is biodistribution. Fluorescent signal was detected in the pancreatic lymph nodes, spleen and pancreas, supporting a contribution to an effective local and systemic effect after tolerogenic antigen presentation. The signal was also detected in the mediastinal lymph nodes, suggesting that liposomes may translocate to the thymus, thus contributing to central tolerance as described after i.p. administration of nanoparticles [47]. The high amount of signal in the perigonadal adipose tissue can be due to liposome affinity for this tissue. No signal was observed in lungs and heart. These results, obtained 24 h after i.p. administration, suggest that signal can be predominantly from phagocytes that have captured PS-liposomes because liposome size and PS promote efferocytosis. In fact, *in vivo* experiments demonstrated that PS-liposomes are captured by phagocytes in 3 hours [15], and the uptake is PS-dependent. Bioimaging results were as expected after i.p. administration of large PS-liposomes [48, 49] and agree with previously reported data in the biodistribution of intraperitoneally injected DCs from NOD mice that migrate to pancreatic lymph nodes, remaining for up to one week [50]. However, additional experiments should be performed to go in depth in this matter.

*In vivo* experiments demonstrated that PSAB-liposomes decrease T1D incidence and slightly delay the onset of the disease. However, no effect was observed when NOD mice received empty PS-liposomes. Empty liposomes display anti-inflammatory effects [15] that fit well with our results in terms of costimulatory molecules expression, anti-inflammatory mediators and T cell proliferation. However, they had no effect in T1D incidence, because the arrest of the autoimmune attack is an adaptive immunity mechanism induced through tolerogenic antigen presentation. This antigen specificity is well supported by our previous study using apoptotic cells with irrelevant antigenic content for T1D [7]. As expected, the prevention of T1D by PSAB-liposomes correlates with the reduction of insulitis, keeping more than 50% of the islets with β-cell mass preservation. In this sense, the here reported results are similar to those obtained with apoptotic β-cells [7] engulfed by DCs. We are conscious of the relevance of the disease stage for a successful immunointervention. In this sense, liposome-based therapy could be optimized by treating NOD mice at earliest stages of the autoimmune attack, during the recruitment of innate cells to the islets and the initiation of diabetogenic T cell response [51]. This stage-from 4 weeks of age- is characterized by a type 1 interferon signature, and can be the best time to start immunotherapy [52].

The MHC class II tetramer analysis was performed as a tool for direct identification of antigen-specific CD4^+^ T cells expanded by tolerogenic DCs. The results confirm that the administration of PS-liposomes loaded with 2.5mi peptide resulted in a fast expansion of antigen-specific CD4^+^ T cells, which is typical for a memory response [53]. No effect was observed when mice were treated with empty PS-liposomes or with 2.5mi peptide. As expected, PS-free liposomes loaded with peptide induced a lower response of antigen-specific CD4^+^ T cells than PS-liposomes in PLN and MDLN, emphasizing the importance of PS as an active component of the liposome in the modulation of antigen-specific CD4^+^ T cell expansion. It is well known that PS is the main ‘eat me’ signal in the engulfment process, but also acts as a ‘tolerate me’ signal in phagocytes [54]. Because PS-liposomes with autoantigens prevent T1D, it is reasonable to speculate that the resulting expanding CD4^+^ T cells have a regulatory function, as otherwise the disease should rather be accelerated. In fact, the percentage of antigen-specific CD4^+^ Foxp3^+^ T cells was moderately increased in mice treated with PS-liposomes loaded with 2.5mi peptide, when compared to sham group. However, since CD4^+^ Foxp3^-^ T cells also expanded, we do not discard that another subset of antigen-specific Foxp3^-^ regulatory T cells [55] could contribute to the effect of this immunotherapy in the induction of tolerance.

The administration of PS-liposomes filled with autoantigens represents a novel approach to down-regulate autoimmunity in T1D. We are well aware that the NOD mouse strain has some immunological defects that are not seen in humans [56], but it is an important tool for dissecting tolerance mechanisms and for the design of innovative therapies. PS-liposomes might provide a new system to co-deliver tolerogenic signals and autoantigens, thus inducing immunological tolerance recovery.

## Conclusions

This study demonstrates that liposomes rich in phosphatidylserine displaying insulin peptides induce tolerogenic DCs and impair autoreactive T cell proliferation. This antigen-specific immunotherapy arrests the autoimmune aggression by apoptotic mimicry, thus decreasing experimental T1D incidence. PS-liposomes can offer a solution with design benefits: customizable, large-scale production, stability, and uniformity. Although further research is needed, the clinical relevance of the herein proposed immunotherapy could prove to be very important, as it has translational potential in human diseases that require the reestablishment of immunological tolerance.

## Supporting Information

S1 Fig Viability and purity of T cells used in proliferation assays. Flow cytometry contour plots of cell size and granularity (FSC and SSC), viability (annexin V^-^, 7aad^-^), purity (CD3^+^), and T cell subsets (CD4 and CD8 expression, gated on CD3^+^ T cells) of negative selected T cells from NOD mice spleen.
(TIF)

S2 Fig Cytokine production in T cell proliferation experiments induced by DCs after the capture of liposomes. Levels of IL-6, TNF, TGF-β, IL-10, IL-2 and IL-4 were measured in supernatants from T cell proliferation experiments induced by iDCs, DCs loaded with empty liposomes (PS-lipo) and DCs loaded with liposomes with insulin peptides (PSAB-lipo) in basal conditions (white bars) or after 24 hours with LPS (black bars). Results are expressed as mean±SEM from four independent experiments. Comparisons within each group and between paired maturation conditions were not able to detect significant differences (p<0.05, Wilcoxon test). Values under dotted line are below the standard.
(TIF)

S3 Fig *In vivo* expansion of antigen-specific CD4^+^ Foxp3^+^ T cells 4 days after i.p. administration of PS2.5mi-liposomes to NOD.Foxp3^EGFP^ mice. A) Representative flow cytometry contour plots of the percentage of 2.5mi^+^ CD4^+^ Foxp3^+^ T cells in the spleen, pancreatic lymph nodes (PLN) and mediastinal lymph nodes (MDLN) (gated on CD19^-^, CD8^-^, F4/80^-^, CD11c^-^, PI^-^ and CD4^+^ cells) in the sham group and after the administration of PS2.5mi-liposomes. Left panel: A^g7^/2.5mi tetramer staining. Right panel: control staining with A^g7^/GPI_282–292_ tetramer. B) Percentage of 2.5mi^+^ CD4^+^ Foxp3^-^ T cells (black bars) and percentage of 2.5mi^+^ CD4^+^ Foxp3^+^ T cells (white bars) in the spleen, PLN and MDLN from 3–5 mice after the administration of saline solution or PS2.5mi-liposomes. Results are expressed as mean±SD. Comparisons between groups showed significant differences (*p<0.05, Mann Whitney test).
(TIFF)
