# Supplementary figures and images for: Use of Autoantigen-Loaded Phosphatidylserine-Liposomes to Arrest Autoimmunity in Type 1 Diabetes
Source: PLoS One. 2015 Jun 3;10(6):e0127057. doi: 10.1371/journal.pone.0127057 (PMC4454589; doi:10.1371/journal.pone.0127057)

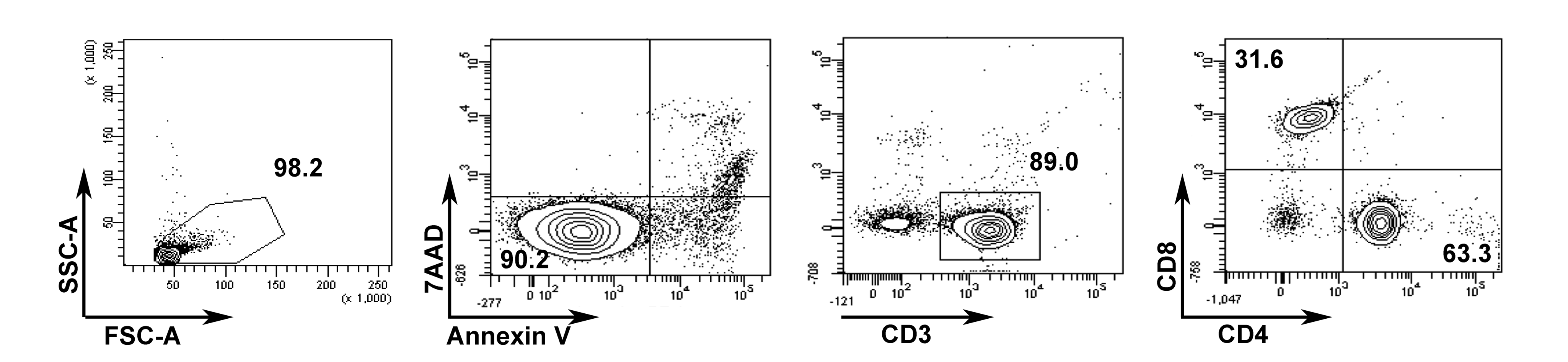

Supplement: S1 Fig — Flow cytometry contour plots of cell size and granularity (FSC and SSC), viability (annexin V-, 7aad-), purity (CD3+), and T cell subsets (CD4 and CD8 expression, gated on CD3+ T cells) of negative selected T cells from NOD mice spleen. (TIF) [file pone.0127057.s001.tif]

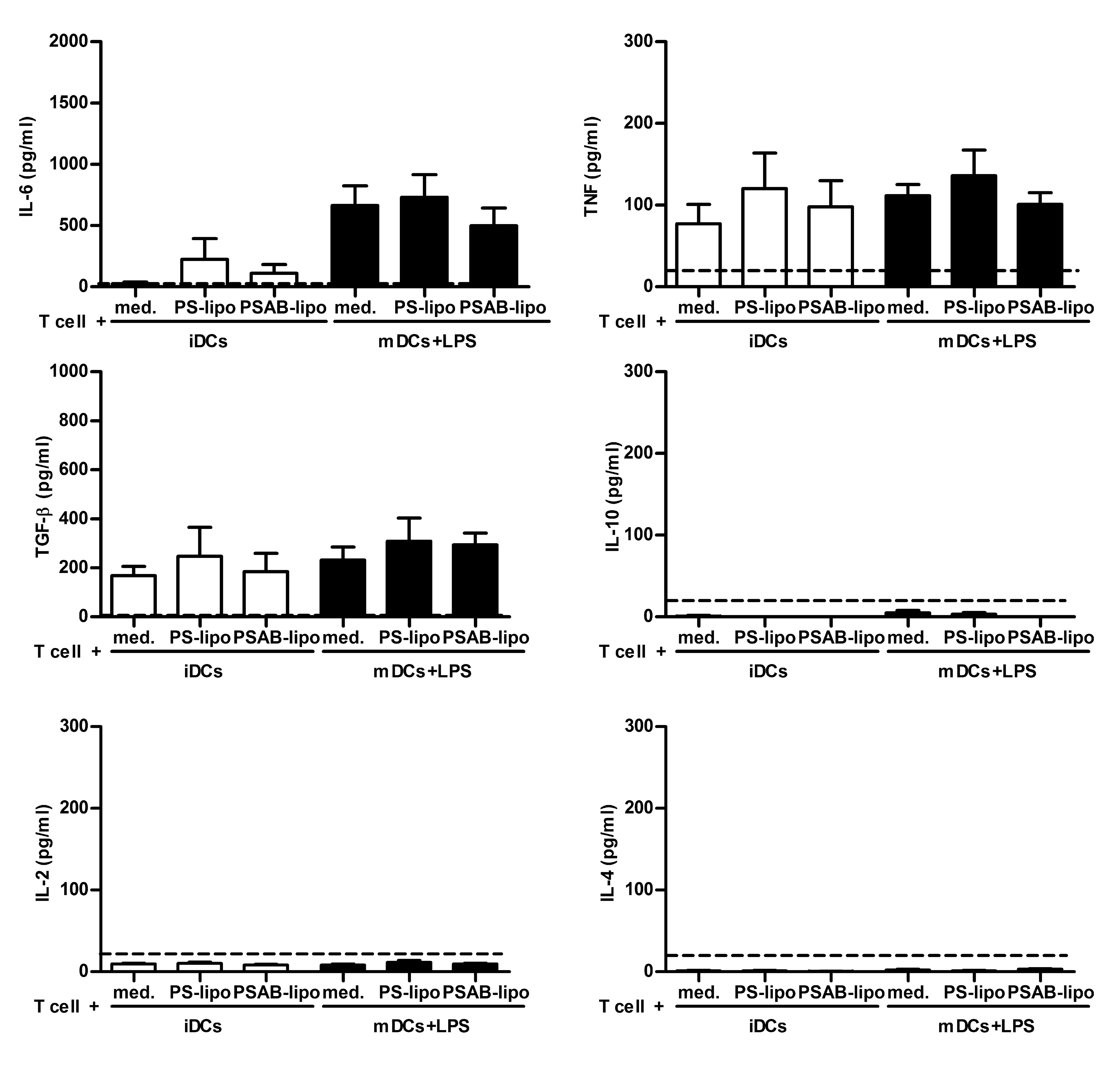

Supplement: S2 Fig — Levels of IL-6, TNF, TGF-β, IL-10, IL-2 and IL-4 were measured in supernatants from T cell proliferation experiments induced by iDCs, DCs loaded with empty liposomes (PS-lipo) and DCs loaded with liposomes with insulin peptides (PSAB-lipo) in basal conditions (white bars) or after 24 hours with LPS (black bars). Results are expressed as mean±SEM from four independent experiments. Comparisons within each group and between paired maturation conditions were not able to detect significant differences (p<0.05, Wilcoxon test). Values under dotted line are below the standard. (TIF) [file pone.0127057.s002.tif]

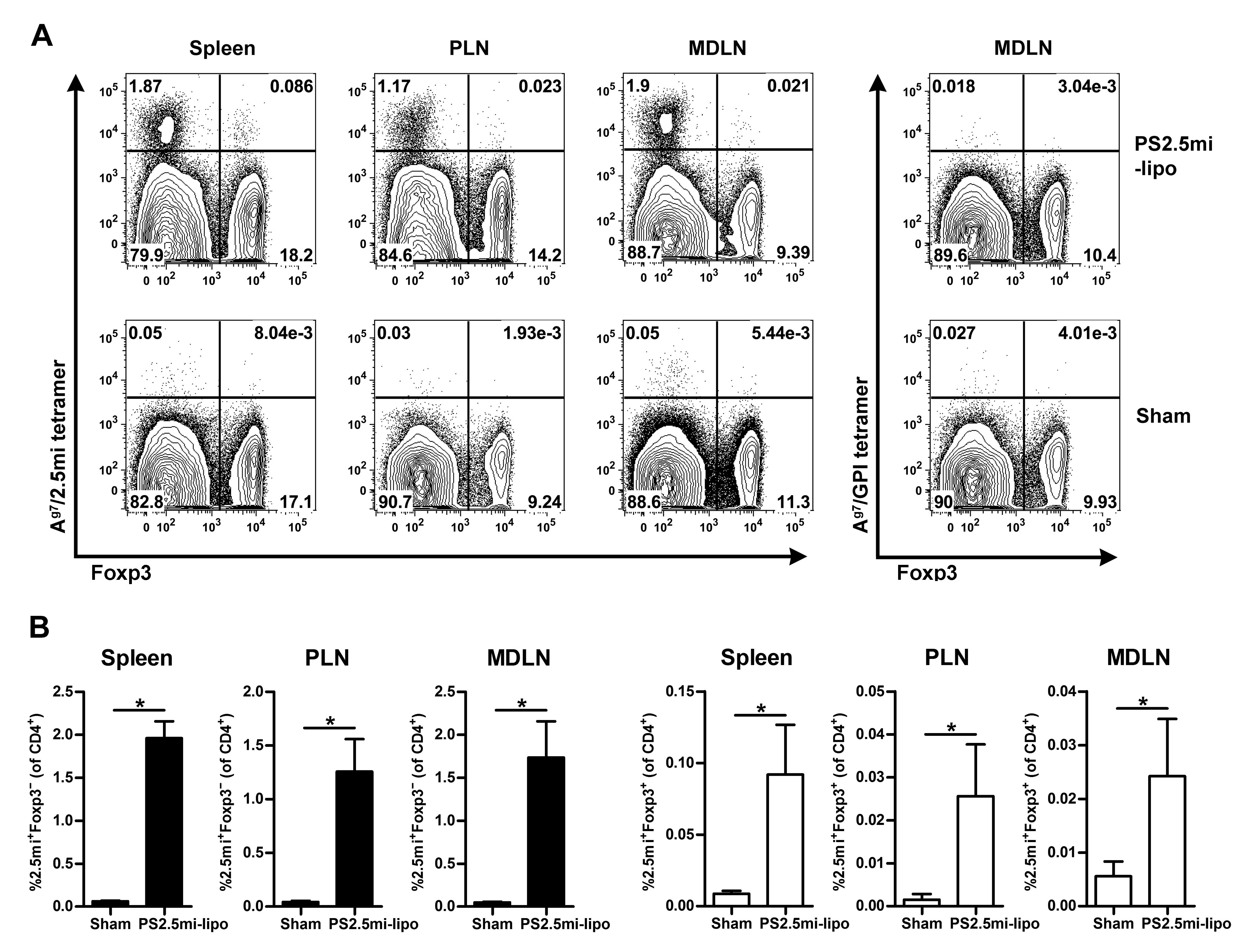

Supplement: S3 Fig — A) Representative flow cytometry contour plots of the percentage of 2.5mi+ CD4+ Foxp3+ T cells in the spleen, pancreatic lymph nodes (PLN) and mediastinal lymph nodes (MDLN) (gated on CD19-, CD8-, F4/80-, CD11c-, PI- and CD4+ cells) in the sham group and after the administration of PS2.5mi-liposomes. Left panel: Ag7/2.5mi tetramer staining. Right panel: control staining with Ag7/GPI282–292 tetramer. B) Percentage of 2.5mi+ CD4+ Foxp3- T cells (black bars) and percentage of 2.5mi+ CD4+ Foxp3+ T cells (white bars) in the spleen, PLN and MDLN from 3–5 mice after the administration of saline solution or PS2.5mi-liposomes. Results are expressed as mean±SD. Comparisons between groups showed significant differences (*p<0.05, Mann Whitney test). (TIFF) [file pone.0127057.s003.tiff]
